# The complete chloroplast genome of *Tanacetum coccineum*

**DOI:** 10.1080/23802359.2020.1768915

**Published:** 2020-06-01

**Authors:** Tuo Zeng, Jiawen Li, Caiyun Wang, Jiefang He

**Affiliations:** a School of Life Sciences, Guizhou Normal University, Guiyang, China; b Key Laboratory for Biology of Horticultural Plants, Ministry of Education, College of Horticulture & ForestrySciences, Huazhong Agricultural University, Wuhan, China

**Keywords:** *Tanacetum coccineum*, chloroplast genome, phylogeny

## Abstract

*Tanacetum coccineum*, a perennial plant of the *Tanacetum* genus, cultivated as a natural pesticide or ornamental plant widely distributed in many countries. In this research, the complete chloroplast genome sequence of *T. coccineum* was determined to comprise a 150,143 bp double-stranded circular DNA, including a pair of 24,416 bp inverted repeat regions (IRs), small single copy (SSC) region of 18,389 bp and large single copy (LCS) region of 82,922 bp. An overall GC content was 37.49%, and the corresponding values in IRs, SSC, and LSC regions are 43.16%, 30.88%, and 35.61%, respectively. A total of 129 genes include 84 protein-coding genes, 37 tRNA, and eight rRNA. Four rRNA genes and seven tRNA genes were duplicated in IRs. A phylogenetic tree reconstructed by 38 Composite family chloroplast genomes sequence reveals that *T. coccineum* is mostly related to *Ismelia carinata*.

*Tanacetum coccineum* (also called *Chrysanthemum coccineum* or *Pyrethrum coccineum*) is a perennial plant of the *Tanacetum* genus which contains more than 160 species of flowing plants, but the extraction of few of them like *T. coccineum* can kill insects. Its product pyrethrin has been widely used as a green insecticide due to its quick knock-down effect on a wide range of insects and low harm to human (Casida 1973). So *T.coccineum* has been employed as the natural pesticide plant in ancient (Ghany 2012). The first cultivated red flower pyrethrum species *T. coccineum*, also called the *Persia pyrethrum*, is native to Caucasus (between the Black Sea and the Caspian Sea) (Contant 1976). ‘Persian Insect Powder’ made from the dried flowers of *T. coccineum* were traded in the first half of the 19th century, which were applied in fighting against ectoparasites such as lice and fleas for children in ancient Persia (Pavela 2016). Although it is common as ornamental plant due to its daisy-like or button-shaped flower heads in shades of white, pink, and yellow, however, its phylogenetic function is still unrecognized due to lack of genome of *T. coccineum*. In this research, we measure the complete chloroplast genome of *T. coccineum* using high throughput sequencing technology, which will provide useful information for the phylogeny of *Tanacetum* genus and further research on the evolution of *Tanacetum*.

The flesh leaves of *T. coccineum* were collected from Flower Nursery Stock Base of Huazhong Agricultural University in Wuhan, China (the specimen was deposited at the Museum of Huazhong Agricultural University accession number: ccau0011589). And the total genomic DNA was extracted from the fresh leaves of *T.coccineum* using Rapid Plant Genomic DNA lsolation Kit (Sangong, Shanghai, China). The whole genome sequencing was conducted by Shanghai Sangong biotechCo., Inc. (Shanghai, China) on the Illumina Hiseq. The filtered sequences were assembled using the NOVOPlasty 2.7.2 (Dierckxsens et al., 2017). Annotation was performed using GeSeq (Tillich et al., 2017). Then, submitted to Genbank (accession number: MT104463).

The cp genome of *T. coccineum* was determined to comprise a 150,143 bp double-stranded circular DNA, including a pair of 24,416 bp inverted repeat regions (IRs), small single copy(SSC) region of 18,389 bp and large single copy (LCS) region of 82,922 bp. An overall GC content was 37.49%, and the corresponding values in IRs, SSC, and LSC regions are 43.16%, 30.88%, and 35.61%, respectively. A total of 129 genes include 84 protein-coding genes, 37 tRNA, and eight rRNA. Four rRNA genes and seven tRNA genes were duplicated in IRs.

To investigate its taxonomic function, 38 Composite family chloroplast genome sequences were aligned by HomBlocks (Bi et al. 2018), and a maximum-likelihood (ML) tree was constructed by MEGAX (Kumar et al. 2018). The species *T.coccineum* was found to be relatively closely related to *Ismelia carinata* compared with other species in Composite (Figure 1). The complete chloroplast genome sequence of *T.coccineum* will provide a useful resource for the conservation family of Composite as well as for the phylogenetic studies of *Tanacetum* genus.

**Figure 1.**
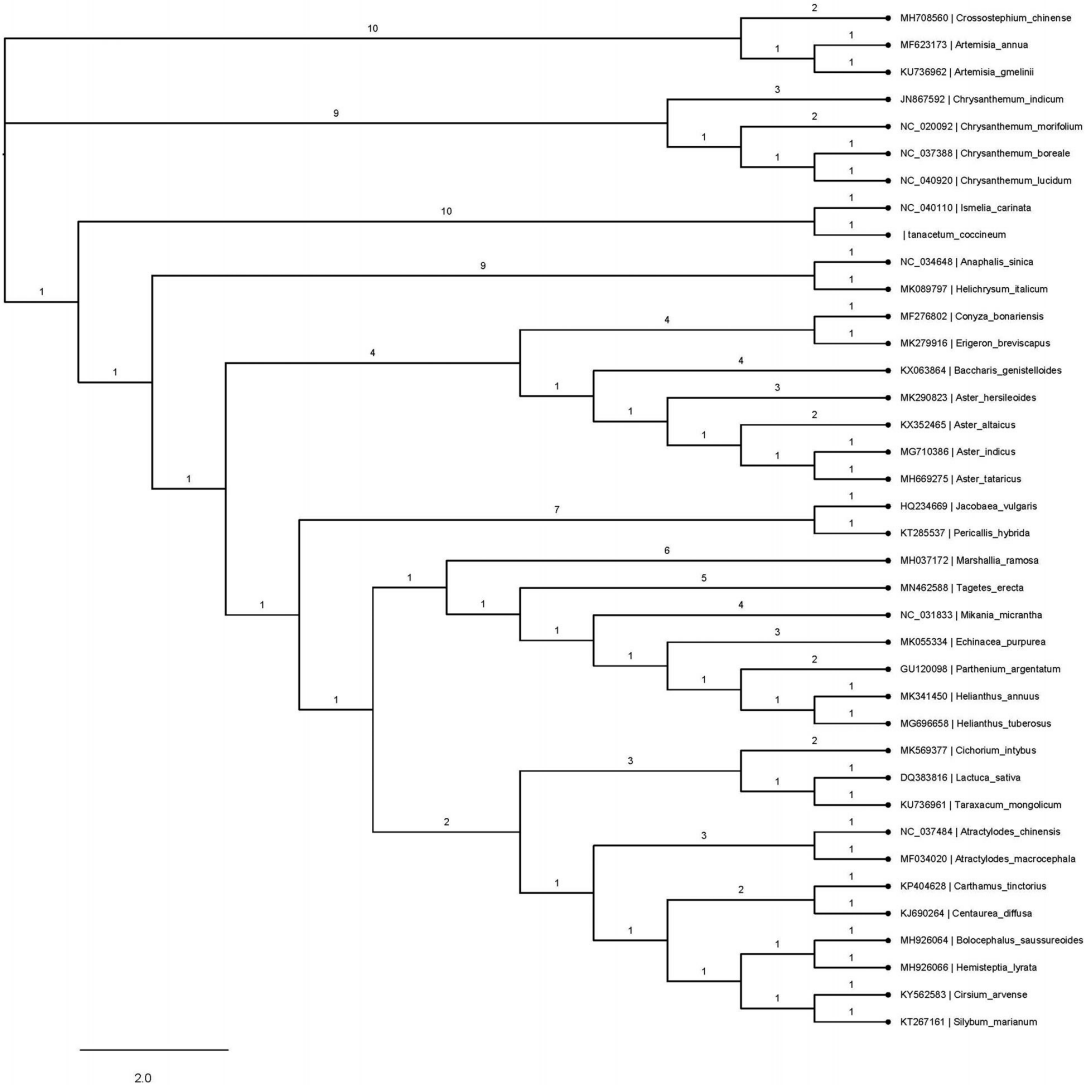
Phylogenetic tree inferred by the maximum-likelihood (ML) method based on 38 representative species. A total of 1000 bootstrap replicates were computed, and the bootstrap support values are shown at the branches. GenBank accession numbers were also shown.

## Data Availability

The data that support the findings of this study are openly available in GenBank at https://www.ncbi.nlm.nih.gov/nuccore/MT104463.1/, reference number [MT104463.1].
